# RETRACTION: Effect of Training Programmes on Nurses' Ability to Care for Subjects with Pressure Injuries: A Meta‐Analysis

**DOI:** 10.1111/iwj.70420

**Published:** 2025-03-26

## Abstract

**RETRACTION:** YanB. , DandanH. , and XiangliM. , “Effect of Training Programmes on Nurses' Ability to Care for Subjects with Pressure Injuries: A Meta‐Analysis,” International Wound Journal 19, no. 2 (2022): 262–271, 10.1111/iwj.13627.34114729 PMC8762546

The above article, published online on 10 June 2021, in Wiley Online Library (http://onlinelibrary.wiley.com/), has been retracted by agreement between the journal Editor in Chief, Professor Keith Harding; and John Wiley & Sons Ltd. Following an investigation by the publisher, all parties have concluded that this article was accepted solely on the basis of a compromised peer review process. The editors have therefore decided to retract the article. The authors did not respond to our notice regarding the retraction.

**RETRACTION:** B. Yan , H. Dandan , and M. Xiangli , “Effect of Training Programmes on Nurses' Ability to Care for Subjects with Pressure Injuries: A Meta‐Analysis,” International Wound Journal 19, no. 2 (2022): 262–271, 10.1111/iwj.13627.34114729 PMC8762546

The above article, published online on 10 June 2021, in Wiley Online Library (http://onlinelibrary.wiley.com/), has been retracted by agreement between the journal Editor in Chief, Professor Keith Harding; and John Wiley & Sons Ltd. Following an investigation by the publisher, all parties have concluded that this article was accepted solely on the basis of a compromised peer review process. The editors have therefore decided to retract the article. The authors did not respond to our notice regarding the retraction.

